# Lung cancer mortality and exposure to polycyclic aromatic hydrocarbons in British coke oven workers

**DOI:** 10.1186/1471-2458-13-962

**Published:** 2013-10-16

**Authors:** Brian G Miller, Emma Doust, John W Cherrie, J Fintan Hurley

**Affiliations:** 1 Institute of Occupational Medicine, Edinburgh, UK

## Abstract

**Background:**

Workers on coke oven plants may be exposed to potentially carcinogenic polycyclic aromatic hydrocarbons (PAHs), particularly during work on the ovens tops. Two cohorts, employees of National Smokeless Fuels (NSF) and the British Steel Corporation (BSC) totalling more than 6,600 British coke plant workers employed in 1967, had been followed up to mid-1987 for mortality. Previous analyses suggested an excess in lung cancer risk of around 25%, or less when compared with Social Class IV (‘partly skilled’).

Analyses based on internal comparisons within the cohorts identified statistical associations with estimates of individual exposures, up to the start of follow-up, to benzene-soluble materials (BSM), widely used as a metric for mixtures of PAHs. Some associations were also found with times spent in certain coke ovens jobs with specific exposure scenarios, but results were not consistent across the two cohorts and limitations in the exposure estimates were noted. The present study was designed to reanalyse the existing data on lung cancer mortality, incorporating revised and improved exposure estimates to BSM and to benzo[*a*]pyrene (B[*a*]P), including increments during the follow-up and a lag for latency.

**Methods:**

Mean annual average concentrations of both BSM and B[*a*]P were estimated by analysis of variance (ANOVA) from concentration measurements at all NSF and six BSC plants, and summarised by job and plant, with a temporal trend (for the BSM only). These were combined with subjects’ work histories, to produce exposure estimates in each year of follow-up, with a 10-year lag to allow for latency. Exposures to BSM and to B[*a*]P were sufficiently uncorrelated to permit analysis in relation to each variable separately.

Lung cancer death risks during the follow-up were analysed in relation to the estimated time-dependent exposures, both continuous and grouped, using Cox regression models, with adjustment for age.

**Results:**

Changing the exposure estimates changed the estimated relative risks compared with earlier results, but the new analyses showed no significant trends with continuous measures of exposure to either BSM or B[*a*]P, nor with time spent on ovens tops. Analyses with grouped exposures showed mixed results. Across all BSC plants, the relative risk coefficient for working 5 or more years on ovens tops, where the exposures were highest, was 1.81, which was statistically significant. However, results for those with 0–5 years on ovens tops did not suggest a trend; the evidence for an underlying relationship was thus suggestive but not strong.

**Conclusions:**

The new results are in line with previous findings; they show some signs consistent with an effect of coke ovens work on lung cancer risk, especially on ovens tops, but the preponderant absence of significant results, and the inconsistencies between results for NSF and BSC, highlight how little evidence there is in these data of any effect.

## Background

Coke is a solid carbonaceous material derived from destructive distillation of low-ash, low-sulphur bituminous coal. It is produced in large ovens in which coal is heated to high temperatures for several hours, driving off volatile and semi-volatile compounds which may include known or suspected carcinogens, to which coke oven workers may be exposed. Among its uses is the reduction of iron ore in the making of iron and steel.

The International Agency for Research on Cancer classify “coke production” as carcinogenic to humans (Group 1) for lung cancer because of exposure to polycyclic aromatic hydrocarbons (PAH) in the industry, and they also note that “although there are no epidemiological studies of benzo[*a*]pyrene, carcinogenicity in many animal species and strong mechanistic evidence justified its classification in Group 1” [1].

The UK Government’s Industrial Injuries Advisory Council has considered the evidence on the risks of lung cancer from work on coke ovens [2]. The strongest evidence is from a large cohort from the USA in which those who worked on coke ovens for more than 15 years had lung cancer risks more than doubled, and doubling of risk was observed for those who had worked for at least 5 years on the ovens tops, where concentrations of PAHs would have been highest [3].

Analyses of coke workers’ mortality in the UK were based on over 6,600 subjects from coke plants operated by National Smokeless Fuels (NSF) and the British Steel Corporation (BSC) [4]. In this cohort the evidence for lung cancer risks was less strong. Based on a 20-year follow-up from 1967, comparisons of over 300 lung cancer deaths with numbers expected using regional rates yielded SMRs of 125% and 127%. However, these reduced to 104% and 110% when compared with rates for Social Class IV (‘partly skilled’), which was considered a more appropriate comparison group for the cohort [5]. For the 1991 report, Hurley *et al.* had updated data on the cohort members’ work histories and obtained and analysed data on job-specific concentrations of benzene-soluble materials (BSM), a measure of the potentially carcinogenic semi-volatile emissions from the ovens. They combined these to estimate individual cumulative exposures to BSM up to the start of the 20-year follow-up, in 1967. Results from regression analyses identified some patterns of excess risk of lung cancer mortality involving these exposures and/or time worked on particular locations (ovens tops, oven sides and elsewhere) in the coke ovens; in general these results were not consistent across the two companies, NSF and BSC. Hurley *et al*. acknowledged that they had not fully used the available data – no allowance was made for risk varying with increases in cumulative exposure during follow-up, which would be relevant for later deaths; and no allowance was made for latency between exposure increment and increased risk of death from lung cancer. They concluded that their report showed sufficient promise that there should be a more comprehensive analysis [5].

We have undertaken a reanalysis of the Hurley *et al*. study that overcomes many of the limitations originally identified [6]. The regression analyses reported here were designed to allow for time-dependency and latency in individuals’ exposures. Data were also available on concentrations of benzo-*a*-pyrene (B[*a*]P), a specific PAH measured as a marker of the mixture, selected because it is relatively abundant in PAH mixtures and from toxicity studies it is known to be one of the most potent carcinogenic PAHs. Analyses were also carried out to see whether conclusions differed when using B[*a*]P exposure estimates as an alternative to BSM.

### Objectives

1. to compare concentration data on BSM and B[*a*]P by plant, job and calendar year;

2. to calculate time-dependent estimates of exposures to BSM and (if warranted) to B[*a*]P;

3. to reanalyse the existing data on lung cancer mortality, incorporating the revised exposure estimates, with suitable allowance for the accumulation of exposure across the follow-up and for latency of cancer outcomes.

## Methods

In the early 1970s, a cohort was established of over 6,600 workers from 13 coke works operated by NSF and 14 BSC plants. They had been employed on 1 January 1967 (NSF) or continuously from 1 January 1966 until 31 July 1967 (BSC). Tracing in British national systems obtained vital status information up to 31 July 1987 for about 98% of the cohort, including cause-specific death data, used as input to SMR calculations [5]. Work histories were collected 1972–77 and updated for continuing workers 1987–88, and were used by Hurley and colleagues to calculate individual exposures up to start of follow-up. Exclusion of those with erroneous data or deficient work histories left 3655 (NSF) and 2707 (BSC) workers for regression analysis of mortality with relation to individual exposures.

We were unable to find the input files for the previous regression analyses, but identified separate cohort vital status and work history files. Following the same procedures yielded an analysis set of 3698 (NSF) and 2707 (BSC) workers with work histories judged as reliable.

Concentration data from the original study [4] had been provided by the companies and used with their permission. These were augmented by records for NSF plants extracted from those publicly available in The National Archives of the UK. These documents provided annual geometric mean concentrations (but no standard deviations) for inhalable dust, BSM and B[*a*]P for job groups at NSF plant, each based on 5-day sampling exercises once or more a year, from 1971 to 1983.

Paper records detailing BSM concentrations in the BSC plants were supplied by their legal successors, Tata Steel Europe, and used with their permission. They were available for only 6 of the 14 BSC plants, covered very limited time periods, and included only a very small amount of measurement data for B[*a*]P. These limited data were entered to MS Excel spreadsheets in the same manner.

Analyses were carried out to identify and quantify the principal sources of variation in the concentration measurements, and to identify and quantify the principal sources of variation in the relationship between B[*a*]P and BSM measurements. Analysis of Variance (ANOVA) on the logarithms of the geometric mean concentrations (with 0.1 added to every value to avoid attempting to take logs of zero) was undertaken using the statistical package GenStat [7], examining the contributions of plant, 14 broad job groups and calendar year. Because not every combination of these factors was represented, the ANOVA was unbalanced, but not seriously so. Similarly analysed were the logarithms of the ratio of BSM to B[*a*]P concentrations. Analyses were restricted to measurements taken from 1975 onwards, once the sampling programme had been piloted and fully introduced.

These analyses produced smoothed concentration estimates by plant, job and year, which were applied to the relevant portions of subjects’ work histories to produce estimates of individual exposures. The data suggested some differences in structure underlying the BSM and B[a]P measurements, respectively. For BSM concentrations at NSF, a time trend estimated at -0.04334 per annum (on the log scale) was applied, allowing forward and backward extrapolation. Concentrations before 1967 were set at the estimated 1967 level. Calculations of exposure combining the estimated concentrations with times worked from work histories [4,5] were programmed using the tabulation and calculation facilities of the statistical package GenStat [7], without and with a ten-year lag to allow for latency. Exposure elements were cumulated over time, to provide cumulative exposure estimates by calendar year for each subject from start of follow-up until the year of death or end of follow-up in 1987.

Exposures to B[*a*]P were calculated similarly; but B[*a*]P concentrations showed no significant time trend, and were therefore not adjusted for calendar year.

Because there were BSM measurements for only six of the BSC plants, and those were very sparse, attempts to derive new summary mean BSM concentrations for the BSC plants and jobs by ANOVA were based on all NSF and BSC measurements, and the predicted concentrations by plant and job group plus a time trend were estimated. These were used with the BSC job histories [4,5] to calculate individual BSM exposure estimates, by year, in the same way as for the NSF plants. B[*a*]P measurements for BSC plants were even sparser, and for only five plants. The mean ratios between B[*a*]P and BSM in the NSF data, by plant and job group, were multiplied by the BSM concentrations estimated for BSC, to estimate annual exposure estimates in the same way. Because the NSF ratios differed somewhat between job groups, the resulting pattern of B[*a*]P exposures by job would be similar but not identical to the same workers’ BSM exposures.

For both NSF and BSC plants, an alternative metric of exposure was calculated based solely on the length of time (years) spent working in broad job groups based on areas of the ovens, specifically Ovens Tops and Ovens Sides; cumulative exposures were calculated time-dependent with a 10-year lag allowance for latency.

Vital status data available had been provided for the original study [4] by UK registration agencies. Cohort flagging had not been maintained in the national registers, and so it was not possible to extend the follow-up period. Analyses of the relationships of risk of death from cancers of the lung, bronchus and trachea - ICD code 162, 8th revision [8] - with cumulative exposures used a Cox proportional hazards framework [9,10], with time since start of follow-up as the underlying time dimension. Data sets merged the data files containing the mortality data – dates and causes of death – with the files of annual cumulative exposure estimates. The regression analyses used the statistical program 2L from the BMDP suite [11]. Analysis adjusted for age, treated as a time-dependent attribute. Subjects’ follow-up was truncated at the earliest of loss from the follow-up, end of follow-up, or death from a different cause.

Results from the regression analyses are quoted as estimated regression coefficients, the ratio of the coefficient to its standard error, and the relative risk obtained by taking the exponential (antilog) of the coefficient.

## Results

Table 1 shows numbers (and percentages) of the cohort members available for mortality analyses here, distributed by decade of birth and by decade of hire, both of which are static characteristics. Analyses with BSM and B[*a*]P exposures used data for members from only 6 BSC plants, and those numbers and percentages are in the last column. All members of the cohorts were male.

**Table 1:** Distribution of cohorts analysed (numbers and %) by decade of birth and decade of hire

**Company**		**NSF**		**BSC (all)**		**BSC (6 plants)**
	**Number**	***%***	**Number**	***%***	**Number**	***%***
**Decade of birth**						
1890 - 1899	0	*0.0*	4	*0.1*	4	*0.3*
1900 - 1909	753	*20.4*	411	*15.2*	188	*12.6*
1910 - 1919	1055	*28.5*	814	*30.1*	419	*28.0*
1920 - 1929	973	*26.3*	855	*31.6*	486	*32.5*
1930 - 1939	586	*15.8*	481	*17.8*	298	*19.9*
1940 - 1949	331	*9.0*	142	*5.2*	102	*6.8*
						
**Decade of hire**						
1910 - 1919	16	*0.4*	8	*0.3*	3	*0.2*
1920 - 1929	94	*2.5*	60	*2.2*	21	*1.4*
1930 - 1939	313	*8.5*	134	*5.0*	41	*2.7*
1940 - 1949	474	*12.8*	267	*9.9*	60	*4.0*
1950 - 1959	1352	*36.6*	1430	*52.8*	881	*58.9*
1960 - 1969	1449	*39.2*	808	*29.8*	491	*32.8*
**Total**	3698		2707		1497	

Smoothed estimates of mean concentrations of BSM and B[*a*]P, standardised to 1978 levels, are given in the Additional file 1. Table 2 summarises the time-dependent exposure estimates calculated using these, for the members of each cohort. We present snapshots of the exposure distributions at the start and middle of the follow-up; exposures later than this are omitted to allow for latency. Distributions are shown separately for those who died of lung cancer at any time during the follow-up period, and those who did not. This was also a dynamic characteristic, with lung cancer deaths occurring throughout the follow-up, intercurrent with deaths from other causes, treated as censoring events. For Table 2 only, death from lung cancer has been treated as a static characteristic, i.e. ignoring when the lung cancer death occurred, and ignoring losses due to deaths from other causes in those who did not die from lung cancer.

**Table 2:** Distribution of cohorts analysed (numbers and %) by exposure metrics (BSM, B[*a*]P, time on ovens tops) grouped, for lung cancer cases and others

			**1967**					**1977**		
	***Lung Cancer***	***%***	***Not Lung Cancer***	***%***	***Total***	***Lung Cancer***	***%***	***Not Lung Cancer***	***%***	***Total***
***NSF***										
**BSM (mg.m**^**-3**^**)**										
Nil	129	*70*	2853	*81*	2982	95	*52*	1973	*56*	2068
0-5	19	*10*	259	*7*	278	19	*10*	588	*17*	607
5-15	15	*8*	188	*5*	203	30	*16*	485	*14*	515
15+	21	*11*	214	*6*	235	40	*22*	468	*13*	508
Total	184		3514		3698	184		3514		3698
**B[ *a *]P (μg.m**^**-3**^**)**										
Nil	129	*70*	2853	*81*	2982	95	*52*	1973	*56*	2068
0-10	18	*10*	302	*9*	320	21	*11*	601	*17*	622
10-30	17	*9*	171	*5*	188	26	*14*	521	*15*	547
30+	20	*11*	188	*5*	208	42	*23*	419	*12*	461
Total	184		3514		3698	184		3514		3698
**Years on ovens tops**										
Nil	162	*88*	3302	*94*	3464	143	*78*	2867	*82*	3010
0-5	19	*10*	150	*4*	169	29	*16*	505	*14*	534
5+	3	*2*	62	*2*	65	12	*7*	142	*4*	154
Total	184		3514		3698	184		3514		3698
***BSC***										
**BSM (mg.m**^**-3**^**)**										
Nil	45	*75*	1087	*76*	1132	29	*48*	549	*38*	578
0-5	9	*15*	204	*14*	213	7	*12*	311	*22*	318
5-15	6	*10*	98	*7*	104	10	*17*	392	*27*	402
15+	0	*0*	48	*3*	48	14	*23*	185	*13*	199
Total	60		1437		1497	60		1437		1497
**B[ *a *]P (μg.m**^**-3**^**)**										
Nil	45	*75*	1087	*76*	1132	29	*48*	549	*38*	578
0-10	8	*13*	218	*15*	226	8	*13*	360	*25*	368
10-30	7	*12*	101	*7*	108	11	*18*	356	*25*	367
30+	0	*0*	31	*2*	31	12	*20*	172	*12*	184
Total	60		1437		1497	60		1437		1497
**Years on ovens tops**										
Nil	110	*86*	2370	*92*	2480	94	*73*	1993	*77*	2087
0-5	16	*13*	184	*7*	200	18	*14*	441	*17*	459
5+	2	*2*	25	*1*	27	16	*13*	145	*6*	161
Total	128		2579		2707	128		2579		2707

Proportional hazards regression models were fitted to lung cancer deaths in various combinations. Predictors were: age, represented by linear and quadratic terms, and cumulative exposure, both calculated as varying throughout the follow-up period and with a 10-year lag to allow for lung cancer latency. Exposures were fitted both as continuous predictors and grouped as in Table 2.

In models fitted to data from NSF plants, the coefficients for linear and quadratic age were statistically significant and similar in magnitude regardless of the exposure fitted. The log-linear coefficient for linear age was around 0.115 per year of age, and the quadratic around −0.0018 per squared year of age. Table 3 includes the estimated coefficient for each model term for exposure, plus its standard error. The exponential (antilog) of the coefficient provides a relative risk estimate, shown here with a 95% confidence interval.

**Table 3:** Results from proportional hazards regression models of lung cancer with time-dependent exposure metrics (BSM, B[*a*]P, time on ovens tops) including 10-year lag

**Variable**	**Units**	**Coefficient**	**Standard error**	**Exp(Coefft) = RR**	**95% Confidence interval**	
***NSF***						
BSM exposure	mg.m^-3^.yr	0.0038	0.0043	1.00	1.00	1.01
BSM 0 – 5	mg.m^-3^.yr	−0.5484	0.3186	0.58	0.31	1.08
BSM 5 – 15		0.3159	0.2061	1.37	0.92	2.05
BSM 15+		0.2017	0.1900	1.22	0.84	1.78
B[*a*]P exposure	μg.m^-3^.yr	0.0006	0.0016	1.00	1.00	1.00
B[*a*]P 0 – 10	μg.m^-3^.yr	−0.3632	0.2777	0.70	0.40	1.20
B[*a*]P 10 – 30		−0.0456	0.2376	0.96	0.60	1.52
B[*a*]P 30+		0.4106	0.1809	1.51	1.06	2.15
Time on tops	yr	0.0113	0.0203	1.01	0.97	1.05
Time on tops 0 – 5	yr	0.3320	0.2161	1.39	0.91	2.13
Time on tops 5+		0.2924	0.2904	1.34	0.76	2.37
***BSC***						
BSM exposure	mg.m^-3^.yr	−0.0158	0.0137	0.98	0.96	1.01
BSM 0 – 5	mg.m^-3^.yr	−0.6041	0.4476	0.55	0.23	1.31
BSM 5 – 15		−0.9863	0.5328	0.37	0.13	1.06
BSM 15+		−0.3353	0.2973	0.72	0.40	1.28
B[*a*]P exposure	μg.m^-3^.yr	−0.0032	0.0062	1.00	0.98	1.01
B[*a*]P 0 – 10	μg.m^-3^.yr	−0.6646	0.4195	0.51	0.23	1.17
B[*a*]P 10 – 30		−0.5882	0.3537	0.56	0.28	1.11
B[*a*]P 30+		−0.3032	0.3565	0.74	0.37	1.49
Time on tops	yr	0.0470	0.0278	1.05	0.99	1.11
Time on tops 0 – 5	yr	−0.0545	0.2642	0.95	0.56	1.59
Time on tops 5+		0.5939	0.2797	1.81	1.05	3.13
						

All models are adjusted for age (linear and quadratic terms).

In the NSF plants, the coefficient for continuous BSM exposure was close to zero, implying a relative risk close to 1 for a unit increase in cumulative exposure, and was far from statistically significant. With the exposures grouped in ranges, with each group compared to those with zero exposure (‘Nil’ in Table 2), there was no evidence that the exposed groups differed systematically in risk from the unexposed group. There was little suggestion of a trend across the exposure groups.

Relating lung cancer risk to B[*a*]P exposures, with continuous exposure, the coefficient was again close to zero, and nowhere near statistically significant. With grouped exposures, at face value the coefficient for the highest exposure suggests a 50% increase in lung cancer risk compared to the unexposed reference group; and a ratio of the estimate to its standard error of 2.27, treated in isolation as a t-statistic, would be statistically highly significant. However, with a grouped exposure it can be misleading to interpret ratios for individual groups as independent t-statistics. Here, the fact that the intermediate groups do not exhibit anything approaching a trend, and the absence of a trend with the continuous exposure, militate against interpreting the high value as evidence of a relationship.

Using as an exposure variable the number of years spent on Ovens Tops, there was no statistically significant relationship with this as a continuous variable. With the time exposure grouped, the middle and high groups appeared somewhat higher than the reference, but the differences were a long way from statistical significance; taken with the continuous result, these are not strong evidence of a real relationship.

A proportional hazards regression model was fitted also to deaths from lung cancer from the six BSC plants for which concentration measurements of BSM were available. There was no evidence of increased risks with continuous BSM exposure; indeed, the coefficient of BSM exposure was negative, though nowhere near statistical significance. With the BSM exposure grouped, again there was no evidence of an increase in risk in the higher exposures groups, with estimated coefficients all negative. Taken together, these tables do not offer any evidence of increased lung cancer mortality risk with BSM exposure. In similar analyses with estimated exposures to B[*a*]P, again there was no evidence of increased risks with higher B[*a*]P.

Analyses in relation to years spent on Ovens Tops used data from all BSC plants. The analysis with continuous exposure produced a positive coefficient, but it was not statistically significant at the conventional 5% level; it was just statistically significant at the 10% level, i.e. P<0.1.

With years on Ovens Tops grouped, the group with five or more years had a coefficient suggesting an 80% increase over the reference group with no such time, and the ratio to standard error of 2.12 would be statistically significant if considered in isolation; however, the intermediate group did not suggest a trend, yielding a negative coefficient, though close to zero and far from statistically significant. The evidence here for an underlying relationship was thus suggestive but not strong.

## Discussion

Cancer risks from work on coke ovens continue to be of interest. Although the steel industry and associated coke-making has declined greatly in the UK, it flourishes in other countries, and any findings from British plants may be relevant elsewhere. In addition, compensation for occupational diseases stemming from historical exposures remains an issue [2], and in that case it is important to have well-quantified risk estimates, not least because of the general requirement in the UK and elsewhere that risk estimated from epidemiological studies should be demonstrably doubled before compensation is applicable.

The extensive reviews by IARC [1] of studies of workers exposed to coal tar and its constituents, including coke plant workers, leave little room for doubt over the carcinogenic potential of the exposures. The meta-analyses of Armstrong *et al.*[12] produced similar relative risk coefficients for similar exposures in three industries of coke ovens, gas-works and aluminium production, at around 1.2 for a lifetime cumulative exposure of 100 μg/m^3^ B[*a*]P. The present results for B[*a*]P exposure fitted as a continuous term (Table 3) yield an equivalent estimate of 1.06 for NSF plants, which lies within that range; for BSC the estimate is 0.73, which is below that range.

However, given the strength of evidence elsewhere, it’s not clear why the present results, based on detailed work histories in both companies and (at least in NSF plants) concentration measurements, are not more positive. The previous studies of coke workers in Britain, including that of Hurley *et al.*[5], have been considered important precisely because they were of reasonable size (cohort numbers and follow-up period), they included all relevant plants from the two major companies in Britain, they had the benefit of both detailed work histories and a reasonable series of concentration measurements, and there was a clear hypothesis arising from earlier US research [13]. That combination allowed Hurley *et al.*[5] to construct individual estimates of both time worked in particular jobs and of cumulative exposure to PAHs measured as benzene-soluble materials (BSM); and to analyse their data on lung cancer mortality in relation to those individual exposures. The exposures used there were not updated through the follow-up period, and the cumulative exposures estimated at the start of follow-up did not allow for the latency expected in lung cancer cases, usually allowed for via lagged exposures. The present results have been quoted with both of these innovations, because we prefer this combination of assumptions. We have also, for comparison, performed and shown the same analyses in terms of exposures to B[*a*]P, having checked that our estimates of annual average BSM and B[*a*]P, by plant and job type, differed sufficiently that separate analyses were meaningful. We noted that only BSM concentrations showed a clear decreasing trend over time, not seen with B[*a*]P. We surmise that this might be because of the relatively low B[a]P concentrations and issues with the limit of detection in the chemical analysis obscuring any trend, but have no data on which to test this.

Davies *et al.* compared the levels of BSM in seven coke oven job categories from the USA and BSC ovens [14]. The arithmetic average levels from the USA were about twice those in the UK. The lower levels of exposure in the British industry may therefore contribute to the lower observed risk compared to other epidemiological studies of coke workers’ mortality.

Exposure misclassification is a potential problem in any study. We had available a much more comprehensive set of BSM and B[*a*]P data for NSF plants than for those of BSC, where only a small number of measurements were taken, and those at only six plants. Consequently, many more men are included in the NSF analyses and the exposure estimates there are more reliable because they are based on measurements (albeit smoothed and, for early years, based on assumption) where those for BSC are largely based on extrapolation from NSF measurements. After omitting the earliest years, for which the data were judged by us as unreliable because they were collected during the development of the sampling and analytical methods, the estimate of the temporal trend in BSM was steeper than in Hurley *et al.*[5] (20% decline versus 13% decline per five years). As a result, concentration estimates for earlier years were increased somewhat.

All the analyses adjusted for age at time of event showed strong effects of age on lung cancer risk. The linear and quadratic age coefficients were similar across NSF and BSC and varied little in models with different exposure metrics. All analyses adjusted for age at time of event.

Taking account of how exposure increases through the follow-up, and including a 10-year lag, the results from the NSF plants showed no convincing evidence of risk increasing with length of time spent in Ovens Tops jobs, or with either BSM or B[*a*]P cumulative exposures. The coefficient for BSM exposure was close to zero, i.e. with a relative risk close to 1, and was far from statistically significant; similarly with B[*a*]P. In grouped analysis there was some, weak, evidence of somewhat higher relative risks in the higher exposure groups. The only result that was formally statistically significant was a higher coefficient for the highest category of B[*a*]P exposure in the grouped analysis (Table 3) but the absence of any evidence of a trend allows also the alternative possibility this may simply be a chance finding.

The results for the BSC plants similarly showed no evidence for a relationship of lung cancer risk with exposure to BSM or B[*a*]P, with coefficients that were actually negative but not statistically significantly different from zero. Those results are based on only the six BSC plants for which concentration measurements were taken, but the modelled concentrations used depended strongly on extrapolation from NSF measurements; and only 60 lung cancer deaths (compared with 184 from NSF). Since work histories were available for all BSC plants, it was possible to include all 14 in analyses with respect to years spent in Ovens Tops jobs. Here the coefficient for 5 or more years in ovens, estimating a relative risk of 1.81, in isolation would be considered statistically significant, but the evidence for an overall trend is of borderline statistical significance. In summary, evidence for an effect was least evident where our exposure data were strongest.

These results, and the estimated relative risk coefficients, differ somewhat from those shown by Hurley *et al.*[5], and this was to be expected, as the methods varied in three ways. The first was in the revised estimates of summary concentrations from the annual average concentrations, where omission of data from years before 1975 increased the coefficient summarising the pattern of concentration decrease with time, within NSF (but not BSC) plants. This would have increased the estimates of earlier exposures up to and shortly after the start of follow-up; other things being equal, a systematic increase in estimated exposures would decrease the relative risk estimate. The second variation was in the introduction of time-dependent exposures, where the addition of exposure corresponding to working times during follow-up would tend to increase exposure estimates and again tend to reduce relative risks. The third change, the introduction of lags, would act to reduce exposure estimates at any time during the follow-up, and this would be expected to increase the relative risks. We consider the present exposure estimates are more appropriate than those used by Hurley *et al*. [5].

Taken together, and with the external SMR analyses reported previously, these results show some signs consistent with an effect of coke ovens work on lung cancer risk among coke workers in the UK, but the preponderant absence of significant results for the new analyses using the more detailed exposure estimates highlights how little evidence there is of any real effect, and inconsistencies between the NSF and BSC results in time on the most exposed Ovens Tops jobs cast doubt on whether the BSC result should be treated as indicating a real effect. The present results echo the general pattern of the earlier analyses, although the relative risk estimate for 5 or more years in Ovens Tops jobs at BSC plants now reduces a little from 2.10 to 1.81 as a result of the changes in how that exposure metric was calculated.

## Conclusions

• Results from NSF plants showed no convincing evidence of risk increasing with length of time spent in Ovens Tops jobs, nor with estimates of individual exposure to either BSM or B[*a*]P.

• Results from BSC plants showed no evidence for a relationship of lung cancer risk with exposure to either BSM or B[*a*]P.

• As in Hurley *et al.*[5], there was a statistically significant increase in risk in men with more than 5 years spent working in Ovens Tops jobs at BSC.

oThe estimated relative risk for this was 1.81, previously 2.10.

• Overall, there was little evidence of an effect of coke oven exposures on lung cancer risk.

## Competing interests

Tata Steel Europe commissioned and paid for the work from the Institute of Occupational Medicine (IOM), including the article processing fee. They also commissioned a separate piece of work on respirators in the industry, to which JWC and BGM contributed. The work was undertaken on the understanding, standard for IOM research and so agreed before the work was commenced, that the results would be published. The company had opportunity to comment on the draft text, which the authors considered before submission. All the authors declare that they have no other competing interests.

## Authors’ contributions

ED extracted and processed the BSM and B[*a*]P concentration data; JWC directed the organisation of the concentration data and the exposure assessment; BGM led the project and performed all the statistical analyses, and was the main author; JFH contributed information on the cohort’s history and assisted in the interpretation of results. All authors contributed to drafting the text and approved the final version.

## Pre-publication history

The pre-publication history for this paper can be accessed here:

http://www.biomedcentral.com/1471-2458/13/962/prepub

## Supplementary Material

Additional file 1 Estimated concentrations of BSM and B[*a*]P by Plant and Job Group. Click here for file
