# Supplementary material for: Lung cancer mortality and exposure to polycyclic aromatic hydrocarbons in British coke oven workers
Source: BMC Public Health. 2013 Oct 16;13:962. doi: 10.1186/1471-2458-13-962 (PMC3854128; doi:10.1186/1471-2458-13-962)
Supplement: Additional file 1 — Estimated concentrations of BSM and B[a]P by Plant and Job Group. [file 1471-2458-13-962-S1.pdf]

Table A1. Definition of job groups for exposure estimation

Location	Job Group	Group Description
Tops	LC	Lorry Car operator
	OL	Oven Lidsman
	VM	Valvesman
Sides	RD	Ram-side doorman
	CD	Coke-side doorman
	GC	Guide-car operator
	SM	Stamp chargeman
	DM	Dauberman
Elsewhere	RC	Ram car operator
	CC	Coke car operator
	WH	Wharfman
	HT	Heaterman

Table A2 BSM concentrations (mg.m^{-3}) by Plant and Job Group, from ANOVA, standardised to 1978

	Job Group*											
	Tops			Side					Elsewhere			
**Plant**	**LC**	**OL**	**VM**	**RD**	**CD**	**GC**	**SM**	**DM**	**RC**	**CC**	**WH**	**HT**
NSF												
1	2.69	2.97	2.35	1.26	1.44	1.42	1.18	1.13	0.86	0.59	0.66	0.90
2	1.95	2.15	1.70	0.91	1.04	1.03	0.85	0.81	0.62	0.43	0.47	0.65
3	1.76	1.95	1.54	0.82	0.94	0.93	0.77	0.74	0.56	0.39	0.43	0.59
4	2.64	2.92	2.31	1.24	1.41	1.39	1.16	1.11	0.84	0.58	0.64	0.88
5	1.85	2.05	1.62	0.87	0.99	0.98	0.81	0.78	0.59	0.41	0.45	0.62
6	1.47	1.62	1.28	0.69	0.78	0.77	0.64	0.61	0.47	0.32	0.36	0.49
7	2.17	2.40	1.90	1.02	1.16	1.14	0.95	0.91	0.69	0.48	0.53	0.73
8	2.07	2.29	1.81	0.97	1.11	1.09	0.91	0.87	0.66	0.45	0.50	0.69
9	2.09	2.31	1.83	0.98	1.12	1.10	0.92	0.87	0.66	0.46	0.51	0.70
10	1.29	1.42	1.12	0.60	0.69	0.68	0.56	0.54	0.41	0.28	0.31	0.43
11	1.82	2.01	1.59	0.85	0.97	0.96	0.80	0.76	0.58	0.40	0.44	0.61
12	2.37	2.62	2.07	1.11	1.27	1.25	1.04	0.99	0.75	0.52	0.58	0.79
13	1.90	2.10	1.66	0.89	1.01	1.00	0.83	0.79	0.60	0.42	0.46	0.63
BSC												
21	1.23	1.39	1.10	0.60	0.68	0.67	0.57	0.59	0.40	0.29	0.32	0.42
22	2.63	2.98	2.35	1.28	1.45	1.43	1.22	1.26	0.87	0.63	0.67	0.89
23	1.20	1.35	1.07	0.58	0.66	0.65	0.56	0.57	0.39	0.29	0.31	0.41
30	1.24	1.40	1.10	0.60	0.68	0.67	0.57	0.59	0.41	0.30	0.32	0.42
32	1.15	1.30	1.03	0.56	0.63	0.63	0.54	0.55	0.38	0.28	0.30	0.39
33	1.41	1.60	1.26	0.69	0.78	0.77	0.66	0.68	0.47	0.34	0.36	0.48

*See Table A1 for key to Job Groups.

Table A3 B[a]P concentrations (µg.m^{-3}) by Plant and Job Group, from ANOVA, standardised to 1978

	Job Group*											
	Tops			Side					Elsewhere			
**Plant**	**LC**	**OL**	**VM**	**RD**	**CD**	**GC**	**SM**	**DM**	**RC**	**CC**	**WH**	**HT**
NSF												
1	8.97	10.62	9.82	2.84	3.05	2.79	1.84	1.85	1.83	0.92	0.85	1.90
2	8.16	9.65	8.92	2.58	2.78	2.54	1.68	1.68	1.66	0.83	0.77	1.73
3	6.25	7.39	6.83	1.98	2.13	1.94	1.28	1.29	1.27	0.64	0.59	1.33
4	8.51	10.07	9.31	2.69	2.90	2.65	1.75	1.75	1.73	0.87	0.81	1.81
5	5.59	6.62	6.12	1.77	1.90	1.74	1.15	1.15	1.14	0.57	0.53	1.19
6	4.12	4.87	4.51	1.30	1.40	1.28	0.85	0.85	0.84	0.42	0.39	0.87
7	11.65	13.78	12.74	3.68	3.96	3.63	2.39	2.40	2.37	1.19	1.10	2.47
8	10.91	12.91	11.94	3.45	3.71	3.40	2.24	2.25	2.22	1.12	1.03	2.32
9	14.47	17.13	15.83	4.58	4.93	4.51	2.97	2.98	2.94	1.48	1.37	3.07
10	8.32	9.85	9.10	2.63	2.83	2.59	1.71	1.71	1.69	0.85	0.79	1.77
11	8.18	9.67	8.94	2.59	2.78	2.55	1.68	1.68	1.66	0.84	0.77	1.74
12	5.10	6.03	5.58	1.61	1.74	1.59	1.05	1.05	1.04	0.52	0.48	1.08
13	7.42	8.78	8.12	2.35	2.53	2.31	1.53	1.53	1.51	0.76	0.70	1.57
BSC												
21	4.92	6.04	5.56	1.65	1.75	1.57	1.07	1.15	1.03	0.56	0.50	1.12
22	10.53	12.94	11.92	3.54	3.75	3.36	2.30	2.47	2.21	1.20	1.06	2.39
23	4.79	5.88	5.42	1.61	1.71	1.53	1.05	1.12	1.01	0.55	0.48	1.09
30	4.94	6.07	5.59	1.66	1.76	1.58	1.08	1.16	1.04	0.57	0.50	1.12
32	4.61	5.67	5.22	1.55	1.64	1.47	1.01	1.08	0.97	0.53	0.46	1.05
33	5.66	6.95	6.40	1.90	2.02	1.81	1.23	1.33	1.19	0.65	0.57	1.28

*See Table A1 for key to Job Groups.
